# The Late Dr. Anna Kingsford

**Published:** 1888-03-17

**Authors:** 


### THE LATE DR. ANNA KINGSFORD.

KATE LACZBROVIC writes from "The Wilderness," Mitcheldean, Gloucestershire: "I was surprised when reading your kindly notice of the late Dr. Anna Kingsford to see that you say 'she was in every sense a humanitarian.' If you will refer to Nuttall, whom I see you recognise as an authority, you will find only one meaning given, viz.: 'Humanitarian, one who holds that Jesus Christ was a mere man.' Did you mean to say that of her?"

[Nuttall's definition is far-fetched and incomplete. We had no intention of giving any opinion on the late Dr. Anna Kingsford's religious views, of which indeed we know nothing. We used the word "humanitarian" in the sense in which it is usually employed, to signify a person who cherishes humane feelings towards all living creatures.—ED. *T. H.*]

